# Comprehensive landscape of integrator complex subunits and their association with prognosis and tumor microenvironment in gastric cancer

**DOI:** 10.1515/med-2024-0997

**Published:** 2024-07-17

**Authors:** Xiaoxia Tong, Li Ma, Di Wu, Yibing Liu, Yonglei Liu

**Affiliations:** Experimental Research Center, Qingpu Branch of Zhongshan Hospital Affiliated to Fudan University, 201700, Shanghai, China; Experimental Research Center, Qingpu Branch of Zhongshan Hospital Affiliated to Fudan University, 1158 East Gongyuan Road, 201700, Shanghai, China

**Keywords:** gastric cancer, INT subunits, INTS11, prognosis, immune cell infiltration

## Abstract

**Backgrounds:**

The integrator complex (INT) is a multiprotein assembly in gene transcription. Although several subunits of INT complex have been implicated in multiple cancers, the complex’s role in gastric cancer (GC) is poorly understood.

**Methods:**

The gene expressions, prognostic values, and the associations with microsatellite instability (MSI) of INT subunits were confirmed by GEO and The Cancer Genome Atlas (TCGA) databases. cBioPortal, GeneMANIA, TISIDB, and MCPcounter algorithm were adopted to investigate the mutation frequency, protein–protein interaction network, and the association with immune cells of INT subunits in GC. Additionally, *in vitro* experiments were performed to confirm the role of INTS11 in pathogenesis of GC.

**Results:**

The mRNA expression levels of INTS2/4/5/7/8/9/10/11/12/13/14 were significantly elevated both in GSE183904 and TCGA datasets. Through functional enrichment analysis, the functions of INT subunits were mainly associated with snRNA processing, INT, and DNA-directed 5′–3′ RNA polymerase activity. Moreover, these INT subunit expressions were associated with tumor-infiltrating lymphocytes and MSI in GC. *In vitro* experiments demonstrated that knockdown of the catalytic core INTS11 in GC cells inhibits cell proliferation ability. INTS11 overexpression showed opposite effects.

**Conclusions:**

Our data demonstrate that the INT complex might act as an oncogene and can be used as a prognosis biomarker for GC.

## 1 Introduction

Gastric cancer (GC) remains the leading cause of cancer-associated death worldwide [1,2]. Due to low rate of routine screening and high metastatic potential of GC, most patients are diagnosed in the advanced stage, with a median survival time of less than 15 months [3]. The present therapeutic strategies, including radiotherapy, biomarker-directed therapy, surgical resection, immunotherapy, and chemotherapy, are insufficient to improve the survival rates of advanced GC patients [1,4]. Hence, new optimal predictive biomarkers and therapeutic targets are badly required for early diagnosis and to prolong the survival of advanced GC.

The integrator complex (INT) is a polyprotein complex consisting of at least 14 subunits, INTS1 to INTS14, with a molecular weight greater than 1.4 MDa [5]. This protein complex binds to the C-terminal domain (CTD) of RNA polymerase II and exerts biological functions in small nuclear RNA processing [6]. INT was first identified as a complex involved in the formation of U-rich small nuclear RNAs [7]. Notably, given its major role in transcriptional regulation and snRNAs processing, it is feasible that some subunits of INT are also involved in the initiation and progression of human tumors [8]. INTS6 was first identified as a candidate tumor suppressor gene in 1999 because it was absent or highly downregulated in non-small cell lung cancer [9]. However, in colorectal cancer (CRC), Ding et al. observed INTS6 was upregulated in CRC and mediated CRC cells proliferation and metastasis by regulating AKT/ERK signaling pathway [10]. Similarly, INTS7 expression was found to be upregulated in tumor tissues of lung adenocarcinoma patients, compared with the adjacent normal tissues [11]. A previous study revealed that INTS8 participates epithelial-to-mesenchymal transition in hepatocellular carcinoma (HCC) [12]. It also functions as an oncogene in intrahepatic cholangiocarcinoma [13].

On the whole, these studies indicated that the INT subunits may be promising therapeutic targets for a variety of tumors. However, the roles of INT subunits in carcinogenesis have not been fully clarified. In particular, there are few reports about INT subunits in GC. It is well known that the systematic bioinformatics analysis of biological functions is one of the most important methods in cancer research. Therefore, in this study, we analyzed the differential expressions and mutation patterns, biological functions, the association with immune cells, protein–protein interactions, and different prognostic values of INT subunits in GC patients based on public databases. Furthermore, INTS11 is the catalytic core subunit of the INT complex and loss of INTS11 would impair the ability of processing U1 and U2 primary transcripts [14]. Therefore, targeting INTS11 may obstruct the function of INT complex, leading to dysregulation of the target genes, and ultimately affecting GC tumorigenesis. Therefore, in our study, a series of experiments were conducted to explore its potential biological functions in GC cells.

## 2 Methods

### 2.1 The Cancer Genome Atlas (TCGA) data analysis

TCGA is a public cancer genomic project supported by the National Cancer Institute that can be used to analyze differences in gene expression and detect co-expressed genes. The RNA-seq transcriptome data of 375 GC tissues and 32 adjacent normal tissues were obtained from TCGA website (https://www.cancer.gov/ccg/research/genome-sequencing/tcga).

### 2.2 Kaplan–Meier (K–M) survival analysis

To explore the potential prognostic values of INT subunits, we analyzed the relationship between INT subunit expressions and overall survival (OS) based on by K–M plotter website (http://kmplot.com/analysis/).

### 2.3 cBioPortal data analysis

cBioPortal (http://cbioportal.org) is a web-based platform for the detection, visualization, and analysis of large-scale cancer genomics data, including epigenetic, gene expression profiling, and proteomics data. We used cBioPortal (Stomach Adenocarcinoma [TCGA, PanCancer Atlas]) to analyze alterations, copy number alterations in the raw RNA-seq data.

### 2.4 Single-cell RNA sequencing analysis

GC scRNA-seq dataset (GSE183904) comprising 11 normal tissues and 29 gastric tumor tissues was downloaded from GEO database. Quality control was performed using the Seurat R package (V 5.0.1), filtering out cells with poor quality (expressed genes <200 or >7,000, mitochondrial gene content >20%). The remaining expressed gene data were normalized using “LogNormalize” scaling and the top 2,000 variable genes were identified using the FindVariableFeatures function (method = vst). Then, data scaling was conducted using ScaleData function, followed by principal component analysis for dimension reduction. Batch effects were corrected using Harmony package (V 1.2.0). Cell types were identified based on marker gene expressions, such as epithelial cells (EPCAM, CDH1, KRT7), T (CD3D, CD8A, CD4), NK cells (NCAM1), endothelial cells (VWF, PECAM1), fibroblasts (COL1A1, PDGFRA), mast cell (CPA3), B cell (CD19, CD79A), plasma B cell (JCHAIN), and myeloid cell (FCGR3A). Data visualization was performed using the UMAP methods.

### 2.5 Relationship between INT subunits and immune cell infiltration in GC

To investigate the specific relationship of INT subunits with immune cells, we utilized TISIDB website (http://cis.hku.hk/TISIDB/index.php) for evaluating whether INT subunits were related to immune subtypes in GC patients. Furthermore, we applied the MCPcounter algorithm for analysis of the relationship between INT subunits and infiltrating immune cells including endothelial cell, neutrophil, cancer-associated fibroblast, monocyte, macrophage monocyte, T cell, T cell CD8, B cell, NK cell, and myeloid dendritic cell.

### 2.6 Correlational research on INT subunits and microsatellite instability (MSI)

MSI is caused by functional defects in DNA mismatch repair and leads to gene duplication disorder, tumor heterogeneity, tumor progression, causing drug resistance or more immune epitopes, and affect tumor prognosis [15]. MSI score was obtained from the TCGA database. Finally, the results were visualized in the form of box plots.

### 2.7 Cell culture

The GC cell lines HGC-27 and AGS were obtained from Cell Bank of Academy of Sciences (Shanghai, China). These cells were cultured in RPMI-1640 medium (Gibco, Life technologies, USA) with 10% fetal bovine serum. All cells were incubated at 37°C in a humidified atmosphere containing 5% CO_2_. All cell lines were authenticated by short tandem repeat profiling.

### 2.8 Proliferation assay

For cell proliferation assay, 1 × 10^3^ transfected cells were seeded into 96-well plates per well. Then, at the indicated time-point, 10 μL of CCK-8 solution (EpiZyme Inc., Shanghai, China) was added into each well, and the plates were incubated for 2 h at 37℃. The optical density values were measured at 450 nm (OD450) by an enzyme labeling instrument.

In parallel, for colony formation assays, 1 × 10^3^ transfected cells were seeded into six-well plates per well. After 14 days, we fixed the colonies with 4% paraformaldehyde and stained the colonies with 0.1% crystal violet. Subsequently, colonies were photographed and counted to evaluate the colony-forming capability of the transfected cells.

### 2.9 Interference of INTS11 expression in GC cell lines and validation

To knockdown INTS11, two shRNA sequences: shINTS11 #1 and shINTS11 #2 and a scrambled Mock shRNA were chemically synthesized at Sangon Biotech (Shanghai, China). Subsequently, the shRNAs were cloned into pLKO.1-shRNA-puromycin vector and co-transfected with packaging plasmids psPAX2 and pMD2G into 293T cells using Lipofectamine 2000 (Invitrogen). The appropriate concentration of virus was added into tumor cells to generate and screen stable INTS11-knockdown GC cells with 1 µg/mL puromycin for 2 days. Then the cells were used for subsequent assays after verifying the INTS11 expression by quantitative real-time polymerase chain reaction (qPCR) and western blotting. shRNAs sequence targeting INTS11 are summarized in Table A1.

### 2.10 INTS11 overexpression in GC cell lines and validation

To investigate the effect of INTS11 overexpression, GC cell lines were transfected with a recombinant plasmid pcDNA3.1-INTS11 (Sangon Biotech, Shanghai, China), which contains the full length cDNA sequence of human INTS11. Both GC cell lines, HGC-27 and AGS, were transfected with the recombinant plasmids. qPCR and western blotting were used to verify the successful overexpression of INTS11 in these cell lines.

### 2.11 Western blotting

Total protein was extracted using RIPA lysis buffer and separated by 10% SDS-PAGE. Subsequently, proteins were transferred onto PVDF membranes. After blocking for 1 h in 5% BSA solution, the membranes were first treated overnight with primary antibodies: INTS11 (1:1,000; 15860-1-AP, Proteintech) and GAPDH (1:1,000; 81640-5-RR, Proteintech). The following day, membranes were treated with HRP-conjugated secondary antibody at room temperature for 1 h at 1:5,000 dilutions. Protein bands were visualized using enhanced chemiluminescence on a luminescent image analyzer (Bio-Rad, Marnes la Coquette, France).

### 2.12 qPCR

Total RNA was extracted using TRIzol reagent and 1 μg of total RNA was reverse transcribed into cDNA using a PrimeScript RT Reagent Kit (Takara Bio, Nojihigashi, Kusatsu, Japan). qPCR analysis of INTS11 and GAPDH gene was performed on a QuantStudio5 real-time PCR system (Applied Biosystems, Life Technologies, USA) using SYBR Green PCR Kit (Takara Bio, Shiga, Japan) according to the recommended protocol. The primer sequences are summarized in Table A1.


**Ethical approval:** This study was based on guidelines of the Declaration of Helsinki and approved by Zhongshan Hospital Qingpu Branch of Fudan University.

## 3 Results

### 3.1 Expression patterns of INT subunits in GC patients

**Figure 1:**
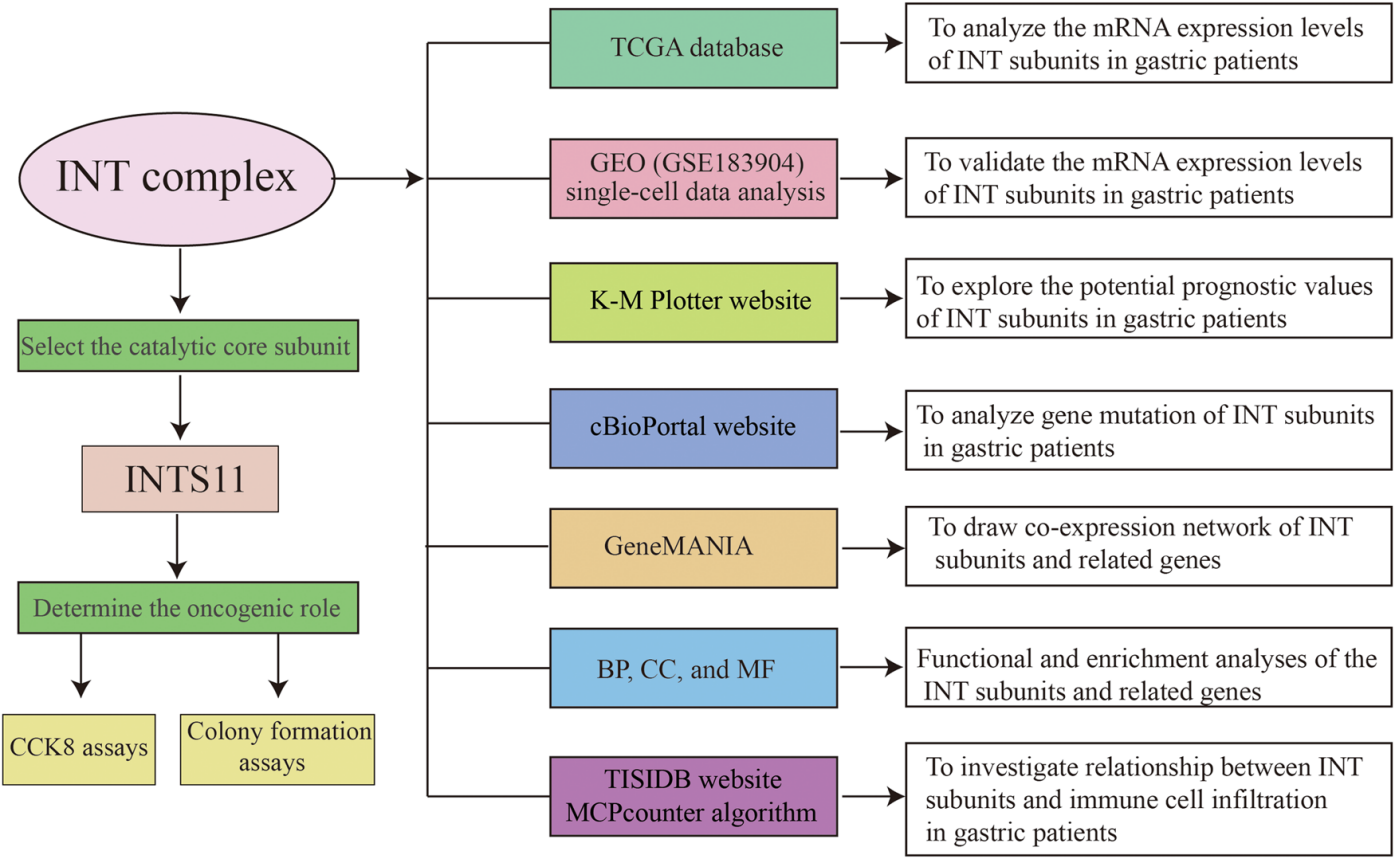
Work flow and the databases used in this study.

**Figure 2:**
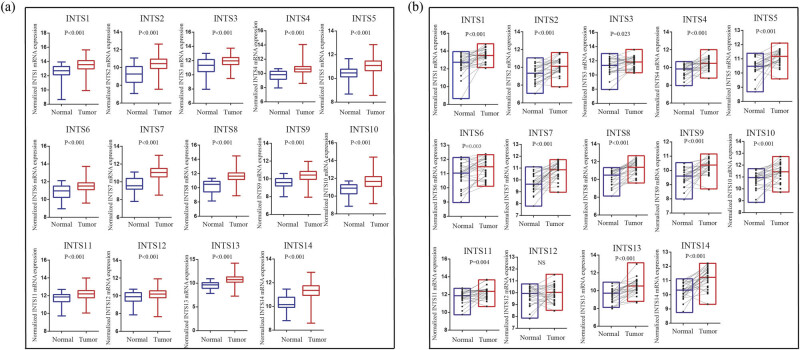
Differences of INT subunits gene expression levels. INT subunits mRNA expression levels were significantly different in the unpaired (a) and paired (b) tumor-adjacent normal GC samples.

**Figure 3:**
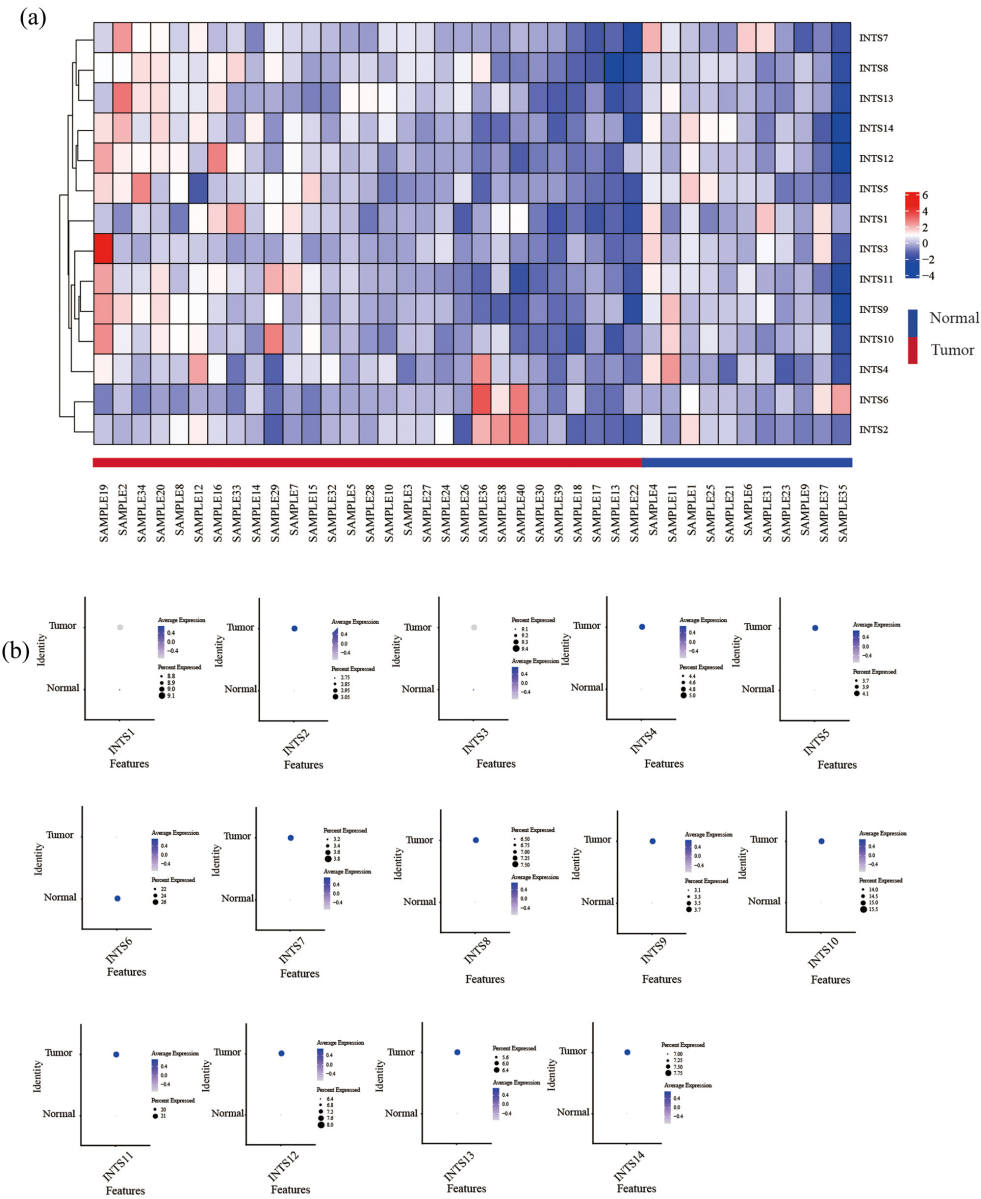
INT subunit expressions in GC tissues and normal tissues were examined by single-cell sequencing analysis. (a) Heat map and (b) dot plot of INT subunit expression profiles identified in gastric normal and cancer tissues.

The research strategy is presented in Figure 1. The TCGA dataset was used to investigate the mRNA expression differences of INT subunits between 375 GC tissues and 32 normal gastric tissues. INTS1–INTS14 expression levels were remarkably elevated in GC samples than in normal gastric specimens, both in unpaired tumor-adjacent normal GC samples and in the paired tumor-normal GC samples (Figure 2a and b). To further verify the expressions of INT subunits in GC patients, single-cell sequencing analysis was conducted. Except for INTS1, INTS3, and INTS6, the heat maps and dot plots showed that the other INTS subunits had higher gene expressions in gastric tissues compared to normal tissues, the heat maps and the dot plots indicate the average expression of the gene in all cell clusters (Figure 3a and b).

### 3.2 Prognostic values of INT subunits in GC patients

**Figure 4:**
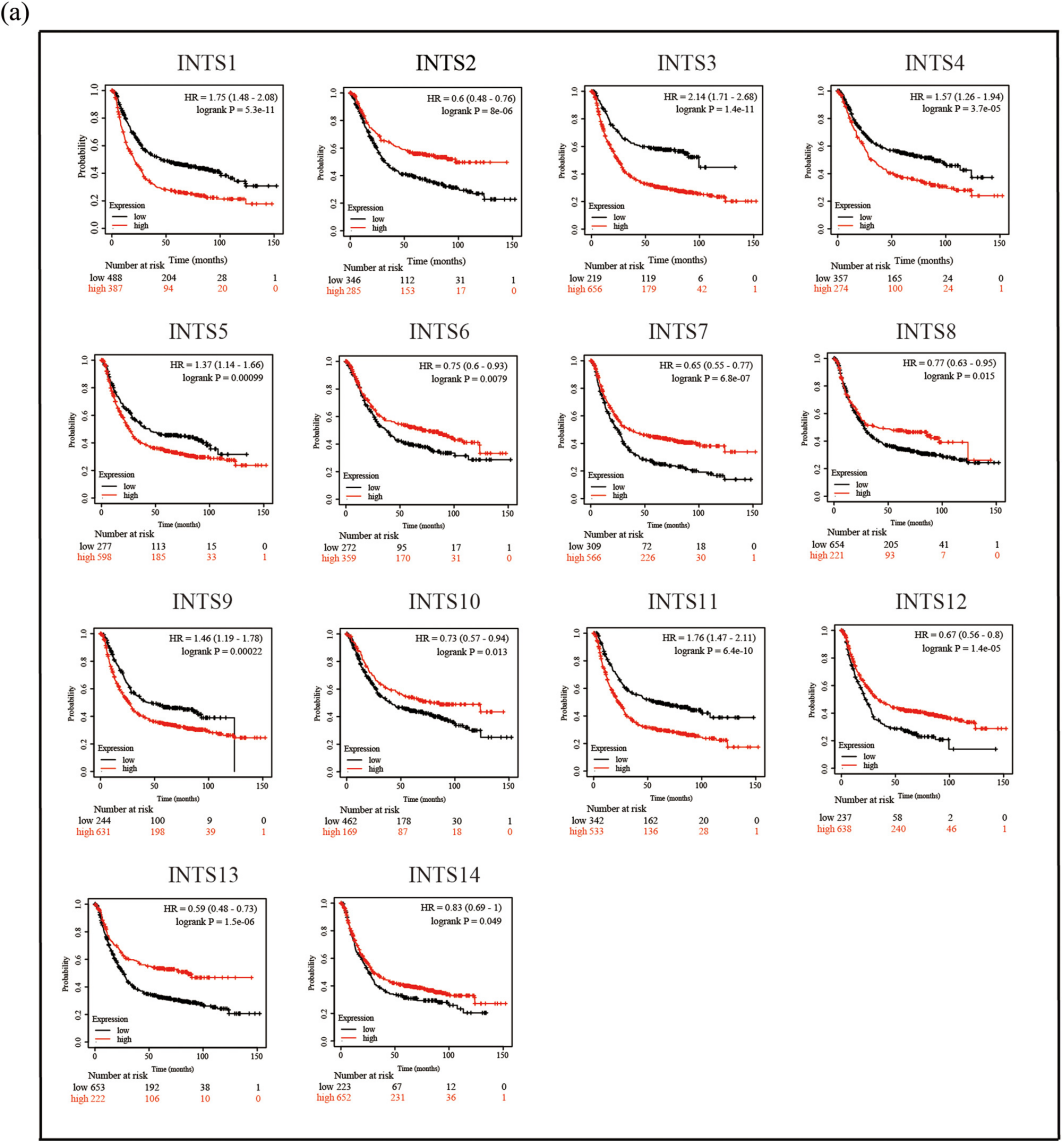
Prognostic analysis of INT subunits in GC patients. K–M analysis of prognostic effects of INT subunits with (a) OS in GC patients. OS, overall survival.

Next, we continued to identify the prognostic values of the INT subunits for GC patients using K–M plotter. The expression levels of INT subunits were divided into high expression group and low expression group according to the best cut-off value provided by the K–M plotter. The K–M curves revealed that higher expression levels of INTS1/3/4/5/9/11 were related to shorter OS (Figure 4a). Conversely, the low expression levels of INTS2/6/7/8/10/12/13/14 indicated poorer OS in all GC patients (Figure 4a).

### 3.3 Gene mutation of INT subunits in GC patients

**Figure 5:**
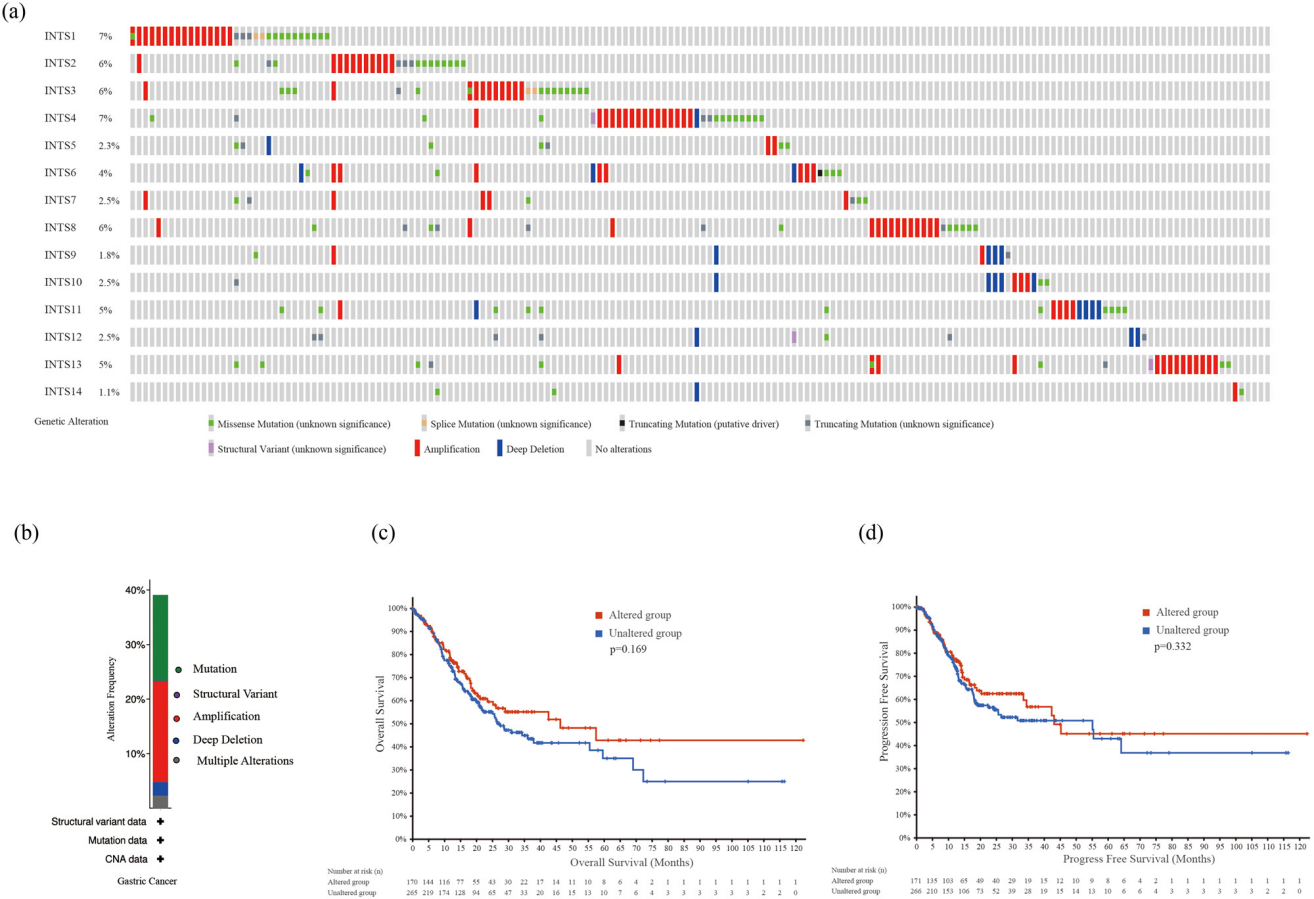
INT subunits gene mutation analysis in GC (cBioPortal). (a) Mutation patterns summary of INT subunits. (b) Mutation frequency of INT complex in GC. (c) K–M plot comparing OS in patients with/without INT subunits gene alterations. (d) K–M plot comparing PFS in patients with/without INT subunits gene alterations. PFS: progression free survival.

cBioPortal website was applied to access the frequency of genetic variation of INT subunits in GC patients. Our findings showed that INTS1 (7%), INTS2 (6%), INTS3 (6%), INTS4 (7%), INTS5 (2.3%), INTS6 (4%), INTS7 (2.5%), INTS8 (6%), INTS9 (1.8%), INTS10 (2.5%), INTS11 (5%), INTS12 (2.5%), INTS13 (5%), and INTS14 (1.1%) were altered in GC samples (Figure 5a). Of the 440 cases, 39.09% exhibited alterations in INT subunits, including mutation (15.68%; 69 cases), structural variant (0.23%, 1 cases), amplification (18.41%; 81 cases), deep deletion (2.5%; 11 cases), and multiple alterations (2.27%; 10 cases) (Figure 5b). In addition, the results of K–M plotter and log-rank test indicated that alterations of INT subunits in GC patients were not significantly associated with OS (log-rank test *p*-value = 0.169) and progression free survival (PFS; log-rank test *p*-value = 0.332) (Figure 5c and d).

### 3.4 Correlation and functional enrichment analysis of INT subunits in GC patients

**Figure 6:**
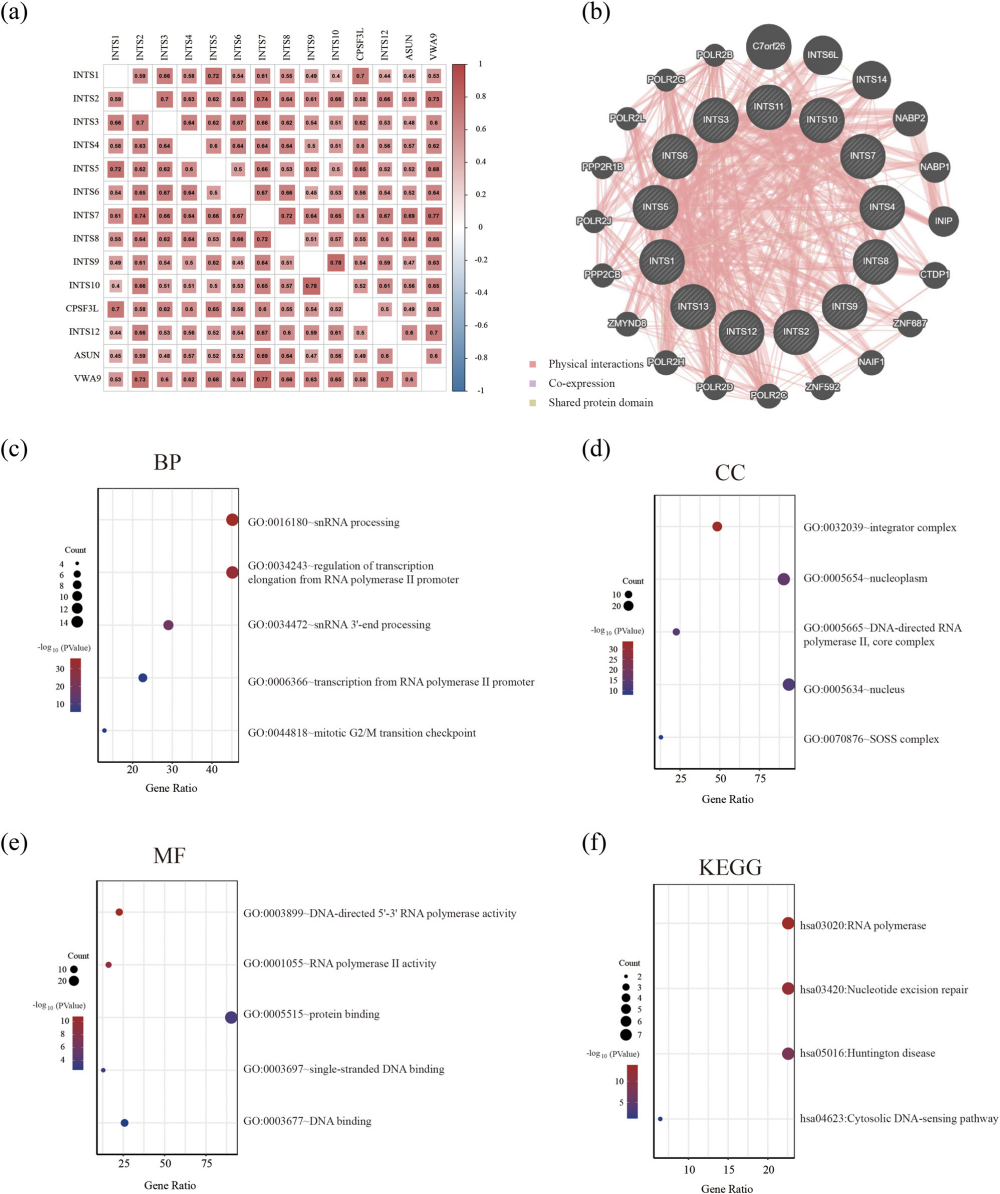
Correlations between INT subunits and related genes functional enrichment analysis in GC patients. (a) Gene correlations between INT subunits were analyzed using the Pearson test in TCGA dataset. (b) Co-expression network of INT subunits and related genes based on GeneMANIA. (c)–(e) Display the top five BP, CC, and MF for INT subunits and related genes, respectively. (f) KEGG pathways of the genes involved. KEGG: Kyoto Encyclopedia of Genes and Genomes; BP: biological process; CC: cellular component; MF: molecular function.

To investigate potential association of INT subunits in GC patients, we performed Pearson correlation analysis to evaluate the correlation between these subunits in GC using TCGA dataset. The results showed that the expressions of these subunits were positively inter-correlated in GC (Figure 6a). In addition, we adopted GeneMANIA to draw gene–gene interaction network between INT subunits and their functionally related genes. The results demonstrated that, except for INT subunits, there were 19 genes, including INTS6L, C7orf26, POLR2B, POLR2G, POLR2L, POLR21B, POLR2J, PPP2CB, ZMYND8, POLR2H, POLR2D, POLR2C, ZNF592, NAIF1, ZNF687, CTDP1, INIP, NABP1, and NABP2, associated with regulatory functions of aberrantly expressed INT subunits in GC patients (Figure 6b). Subsequently, functional and enrichment analyses of the above genes were performed using DAVID and presented in bubble diagrams. We found that the main biological process (BP) of these genes was snRNA processing and the main cellular component (CC) of these genes corresponds to INT (Figure 6c and d). The main molecular function (MF) of these genes was DNA-directed 5′–3′ RNA polymerase activity (Figure 6e). Kyoto Encyclopedia of Genes and Genomes (KEGG) pathway analysis was adopted to clarify pathways related to INT subunits and related genes, and the results revealed that these genes were mainly involved in RNA polymerase (Figure 6f).

### 3.5 Relationship between INT subunits and immune cell infiltration in GC

**Figure 7:**
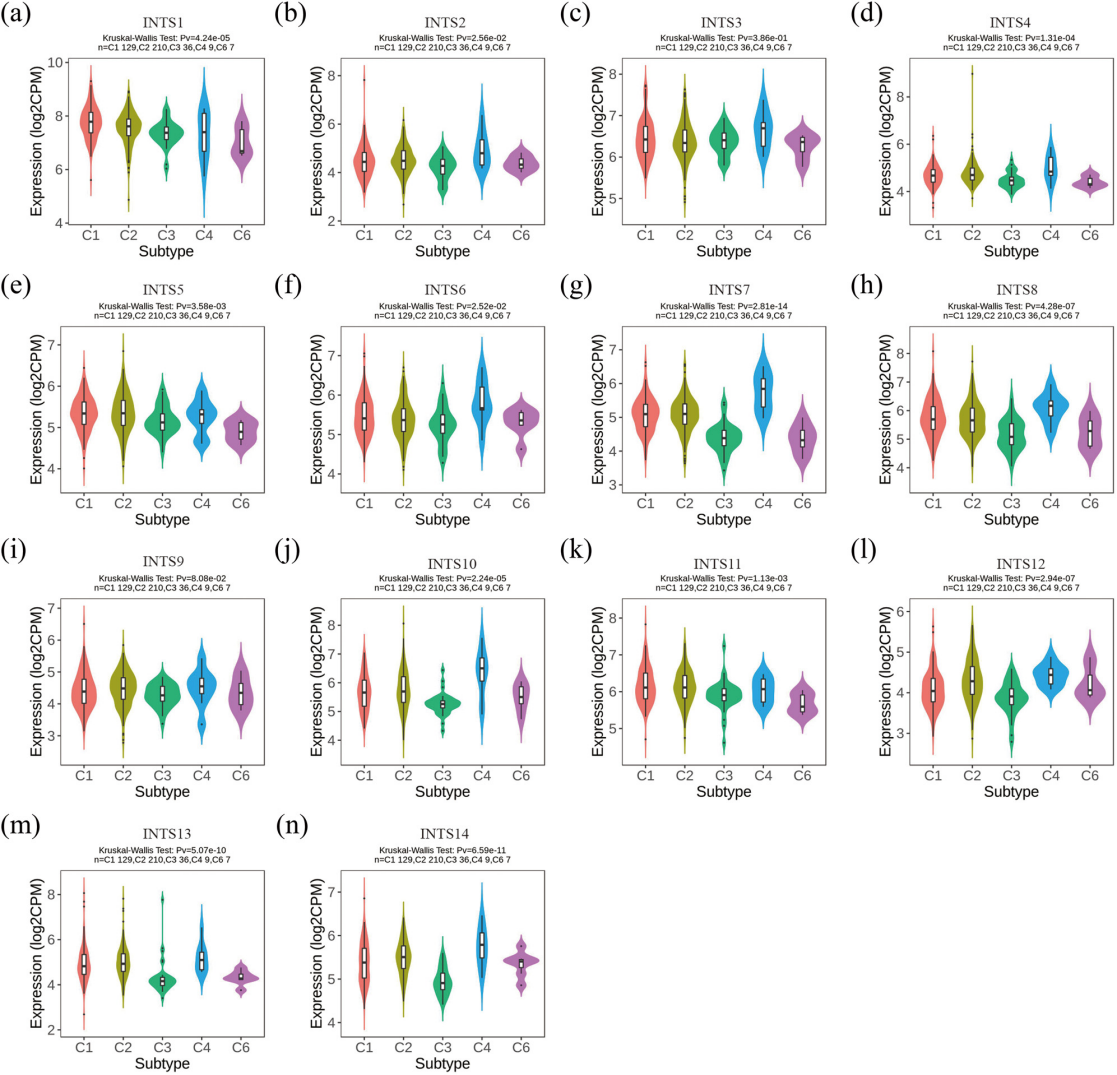
Effect of INT subunits on immunological status in GC using the TISIDB. (a)–(n) INT subunits mRNA expressions in different immune subtypes in GC, C1 (would healing), C2 (IFN-gamma dominant), C3 (inflammatory), C4 (lymphocyte depleted), and C6 (TGF-β dominant).

**Figure 8:**
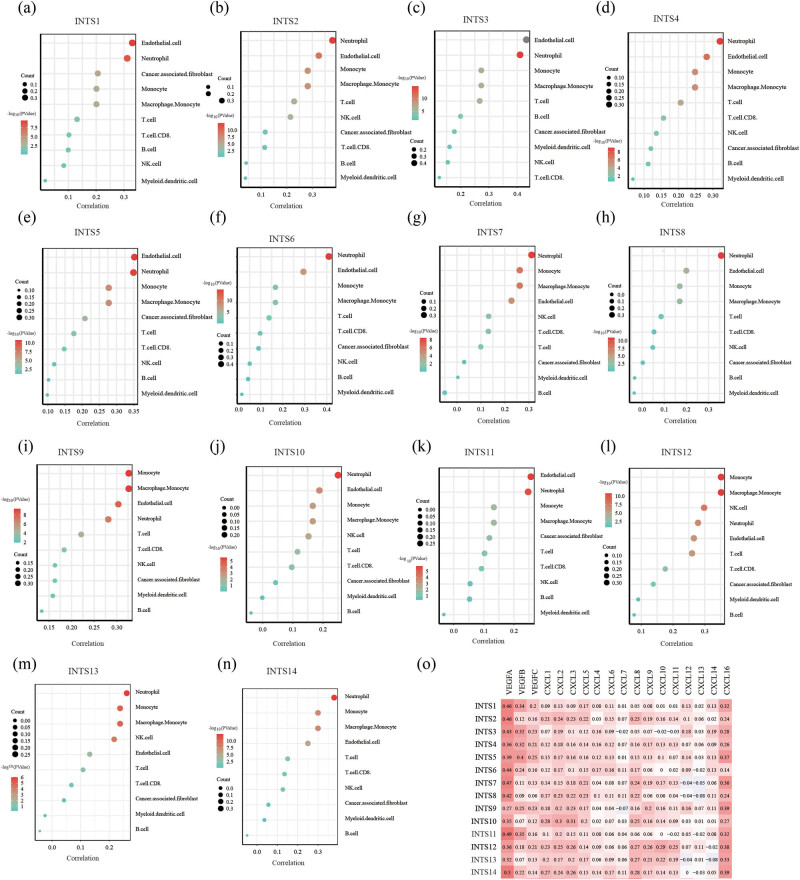
Correlations between the expression of INT subunits and immune infiltrating cells, CXCL chemokines, and VEGFs (a)–(n) Correlations between the expression of INTS11-14 subunits and immune infiltrating cells, respectively. (o) Correlations between INT subunits and CXCL chemokines, VEGFs.

To further explore the expression of INT subunits across immune and molecular subtypes, the TISIDB website was applied to perform an integrated analysis. Interestingly, we found that the expression levels of INT subunits were significantly different across different immune subtypes (Figure 7a–n). Subsequently, we further investigated the association between INT subunit expressions and immune cell infiltration in GC using MCPcounter algorithm. Our results showed that most INT subunit expressions were strongly positively related to neutrophil and endothelial cells (Figure 8a–n). Infiltration and polarization of neutrophils are mainly mediated by ELR + CXC (CXCL) chemokines and regulated by various stimulant-cell interactions [16–18]. So we further verified the correlation between INT subunits and CXCL chemokines and our results indicated the INT subunit expressions were positively associated with most of the CXCL chemokines (Figure 8o). Vascular endothelial growth factor (VEGF) is a special class of cytokines, which is only involved in regulating the biological activity of endothelial cells [19,20]. Functionally, VEGF can specifically induce interstitial production and promote vascular endothelial growth [21]. So we also probed the correlation between INT subunits and three VEGFs. The correlation analysis showed that INT subunits had a strong positive correlation with these three VEGFs (Figure 8o).

### 3.6 Association of INT subunits with MSI

**Figure 9:**
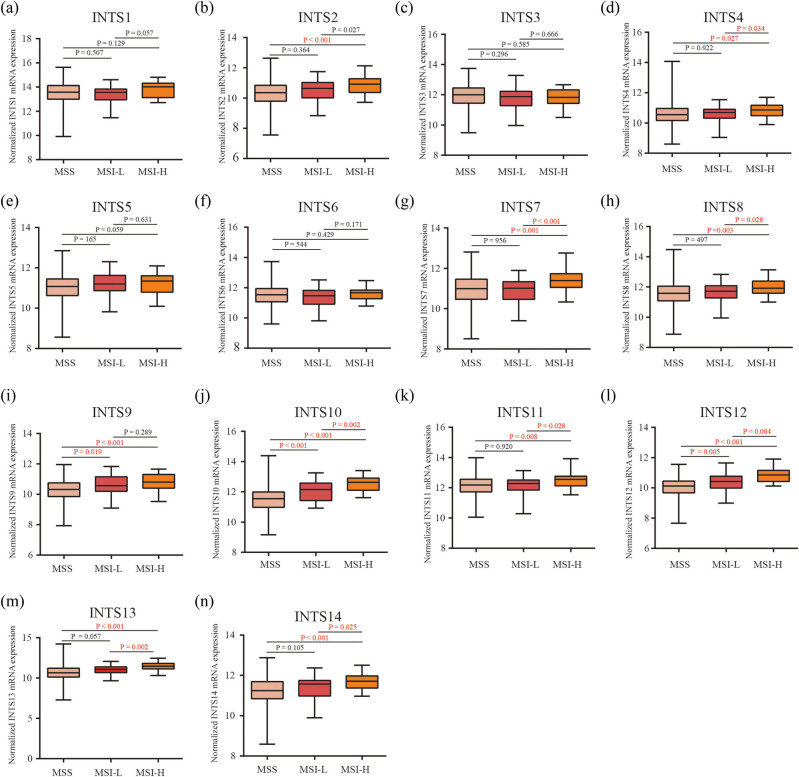
Correlations between MSI and the expression of INT subunit genes. (a)–(n) INTS1–14, respectively. MSI: microsatellite instability.

Numerous studies have demonstrated the value of MSI as indicators of the tumor immune response and linked with immune checkpoint inhibitor sensitivity. We thus performed MSI investigations. Our results found that most of the INT subunit expressions were positively associated with MSI in GC (Figure 9a–n), indicating that INT subunits may play important roles in the immune regulation and could predict the immunotherapy effect in GC. All these data together indicated that most of INT subunit expressions are widely associated with immunity in GC.

### 3.7 INTS11 distribution in GC were examined by single-cell analysis

**Figure 10:**
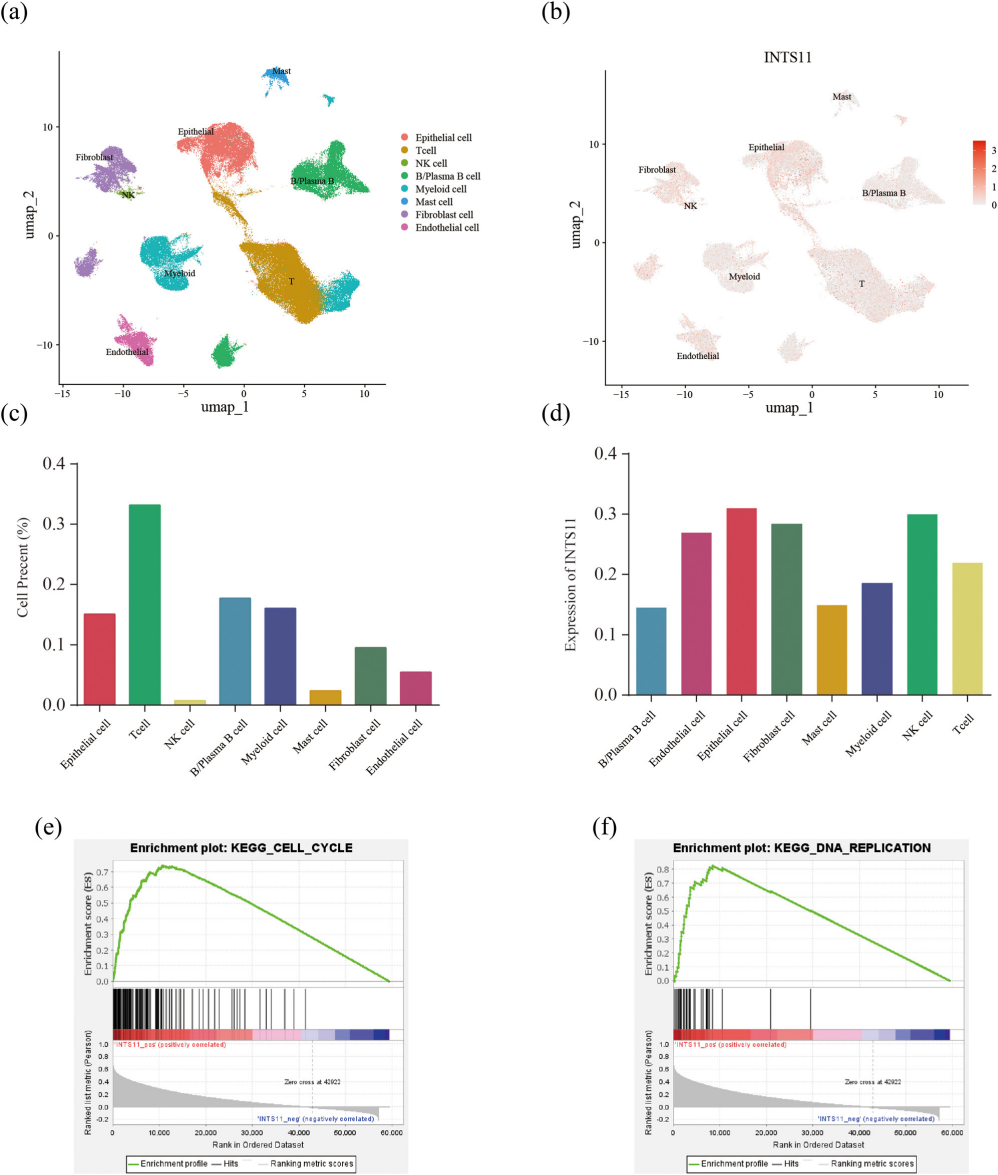
INTS11 expression in different types of cell in GC was examined by single-cell analysis. (a) Cell clusters of GSE183904 of 29 GC patients. (b)–(d) INTS11 expression and distribution in eight clusters of cell types. (e) and (f) Hallmark pathways of tumors associated with INTS11.

**Figure A1:**
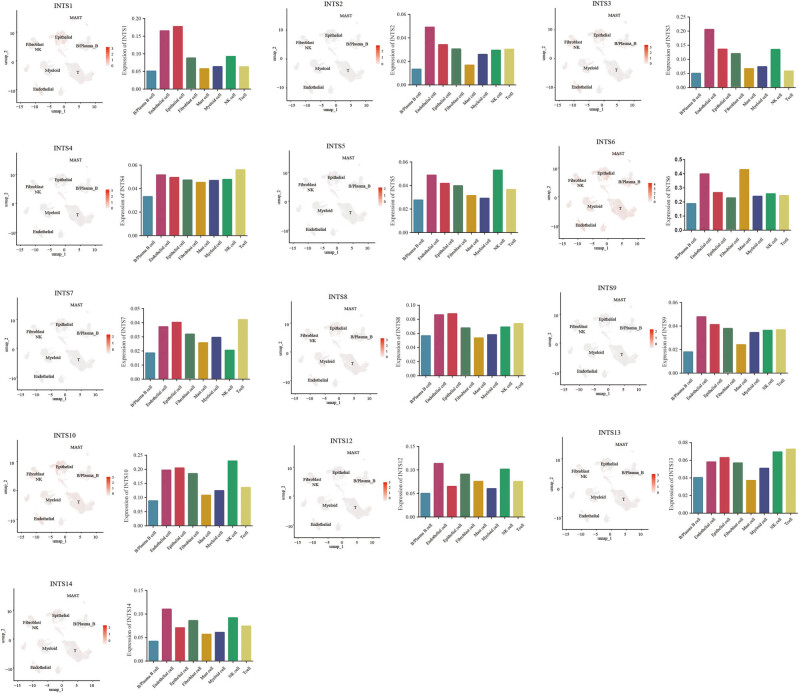
UMAP plots and the expression of INT subunits in different cell types.

INTS11 is the catalytic core subunit of the INT complex and the loss of INTS11 would impair the ability of processing U1 and U2 primary transcripts [22]. It is essential for the eviction of paused RNAPII and transcriptional elongation [23]. Therefore, targeting INTS11 may obstruct the function of INT complex, leading to deregulation of the target genes, and ultimately affecting GC tumorigenesis. To further determine the oncogenic role of INT complex in GC, we analyzed GSE183904 to examine the association between the expression of catalytic core subunit INTS11 and different types of cell. Based on the marker gene expression, we identified eight clusters of cell types, including epithelial cells, fibroblasts, myeloid cells, B and plasma B cells, T cells, mast cells, NK cells, and endothelial cells (Figure 10a). The UMAP plots showed that INTS11 could express in all cell types, especially in epithelial cells (Figure 10b–d). The UMAP plots and the expression of others INT subunits in different cell types are shown in Figure A1. Moreover, GSEA was conducted and found that there were positive correlations between high INTS11 expression and hallmark pathway of tumors, such as DNA replication and cell cycle pathways (Figure 10e and f).

### 3.8 Knockdown of INTS11 inhibited proliferation of GC cells

**Figure 11:**
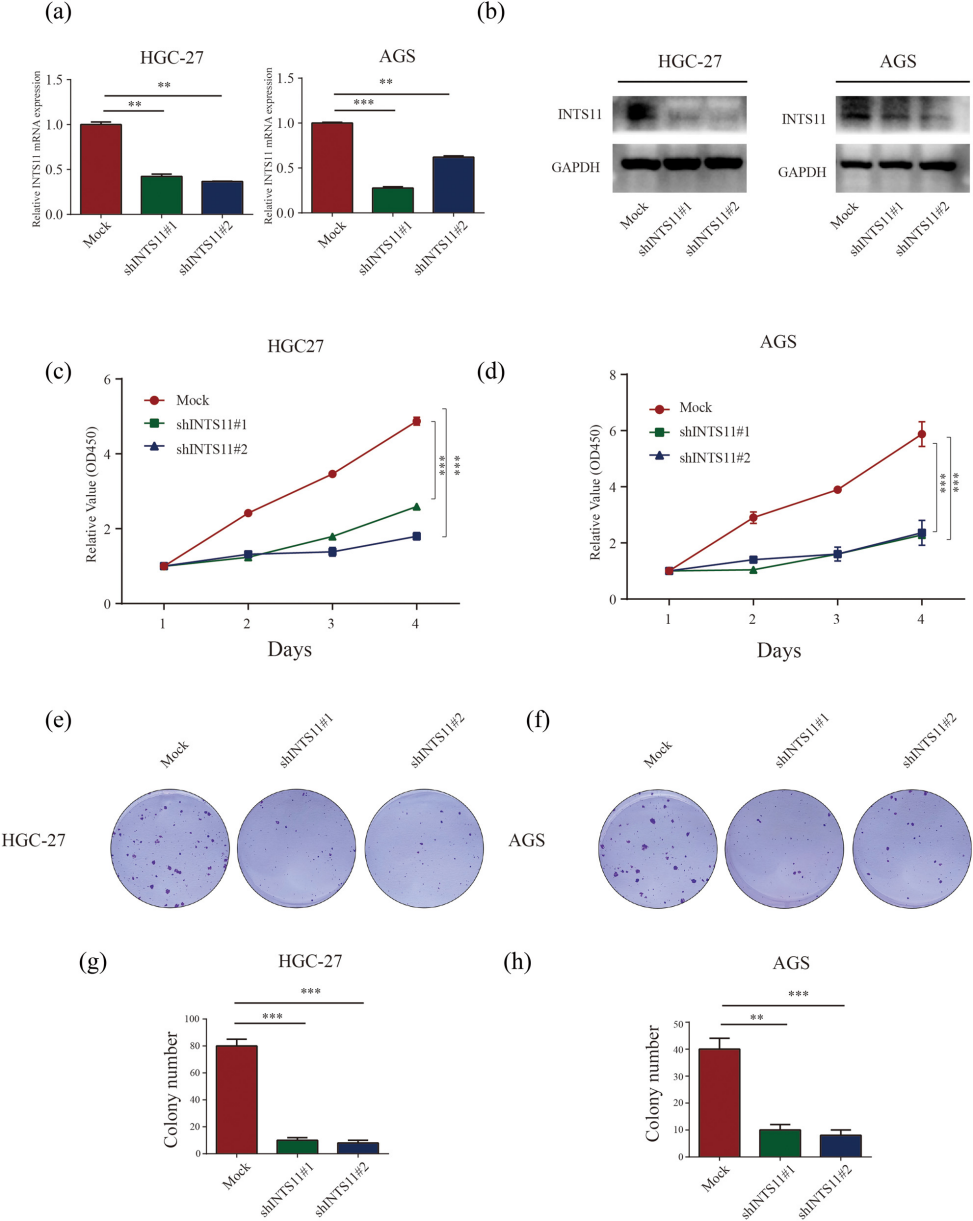
Knockdown of INTS11 inhibited GC cell proliferation. (a) qPCR and (b) western blotting analysis verified successful decreased expression of INTS11 in HGC-27 and AGS cells. (c) and (d) In HGC-27 and AGS cells, CCK8 assays showed knockdown of INTS11 significantly inhibited cell growth. (e)–(h) In HGC-27 and AGS cells, colony formation assays showed knockdown of INTS11 significantly inhibited cell colony formation abilities.

Next, we continued to investigate the role of INTS11 in GC cell proliferation. The result of INTS11 down expression was verified by qRT-CR and western blotting assays independently (Figure 11a and b). Then we performed CCK-8 assays to investigate the role of this complex in GC cell proliferation and the results showed that knockdown of INTS11 significantly inhibited cell proliferation of AGS and HGC-27 cells compared to its corresponding controls (Figure 11c and d). The effect of INTS11 on cell viability was further verified in colony formation assays. Results showed that knockdown of INTS11 produced less colonies compared to the mock groups (Figure 11e–h).

### 3.9 INTS11 promoted cell proliferation in GC cell lines

**Figure 12:**
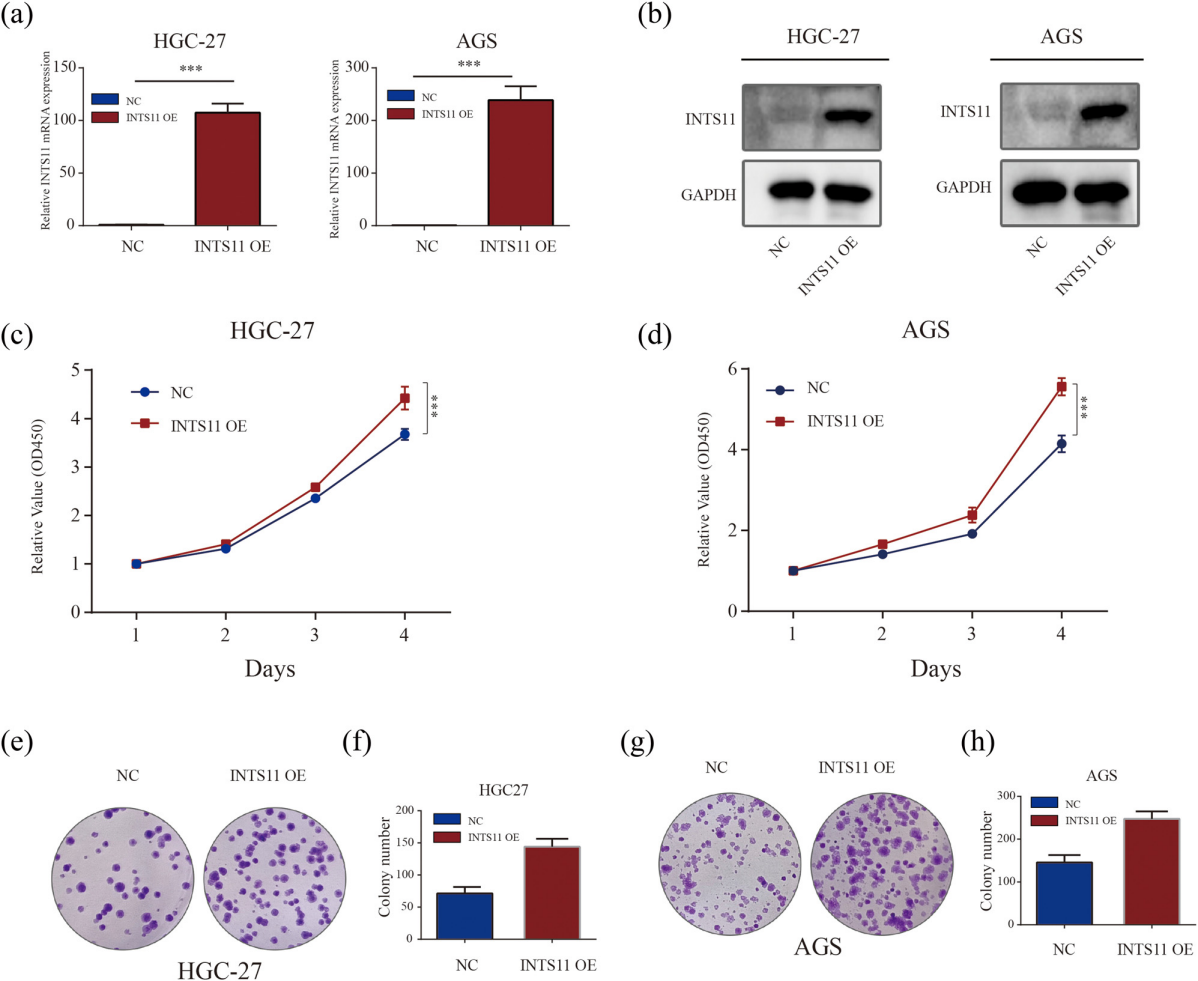
Overexpression of INTS11 promoted GC cell proliferation. (a) qPCR and (b) western blotting analysis verified successful increased expression of INTS11 in HGC-27 and AGS cells. (c) and (d) In HGC-27 and AGS cells, CCK8 assays showed overexpression of INTS11 significantly promoted cell growth. (e)–(h) In HGC-27 and AGS cells, colony formation assays showed overexpression of INTS11 significantly promoted cell colony formation abilities.

We subsequently enhanced the expression of INTS11 protein in HGC-27 and AGS cell lines via transfecting with a pcDNA3.1-INTS11 overexpression vector. The efficacy of INTS11 overexpression was independently confirmed through qPCR and western blotting assays (Figure 12a and b). The CCK8 assays revealed that INTS11 overexpression significantly enhanced the proliferative abilities of HGC-27 and AGS cells (Figure 12c and d). Additionally, the enhanced expression of INTS11 was observed to promote colony formation in these cell lines (Figure 12e–h).

## 4 Discussion

This is the first study to provide a systematic and comprehensive overview of genetic alterations, prognostic values, and the expression patterns of all genes encoding INT complex subunits in GC. INT complex is metazoan specific protein group composed of at least 14 subunits with a variety of biological functions, like impacting the transcriptional activation, small nuclear RNA production and processing, blast lineage development, and nucleic acid metabolism [24].

Many studies have shown that dysregulation of INT subunits is involved in occurrence and development of multiple cancers [7,25]. Although several INT subunits have been shown to play critical regulatory roles in tumors, the distinct roles of INT subunits in GC remain to be elucidated. In this study, bioinformatics tools were applied to identify mutations, mRNA expressions, prognostic values, the association with MSI, and biological significance of different INT subunits in GC.

INT complex is associated with the biogenesis of enhancer RNAs development and small nuclear RNA transcription regulation [23,26]. Teng found that mutations in INTS1 exerted their function during carcinogenesis mainly via post-transcriptional mechanisms [27]. Also, INTS1 gene mutations were associated with rare recessive human neurodevelopmental syndromes [6]. INTS6, also named as DICE1, is a member of the ATP-dependent helicases and related proteins [9]. It showed loss or downregulation of protein expression in the great mass of NSCLC tested [28]. In prostate cancer (PC), hypermethylation of the DICE1 promoter was observed in PC cell lines and in four of the eight tested PC patients [29]. INTS7 was involved in ATR pathway activation to promote viral genome replication [30]. INTS7 also worked with BAG3 to regulate bone marrow mesenchymal stem cell proliferation and migration [31]. As for INTS8, Yin et al. found that it was stably upregulated in HCC and Tong et al. demonstrated that INTS8 can accelerate the epithelial-mesenchymal transition in HCC by participating in the TGF-β signaling pathway [12]. INTS11 was a kind of catalytic nuclease and it was also the catalytic subunit of the INT complex [22]. The structure analysis revealed that INTS11 binds to INTS9 to constitute the catalytic core of INT complex, and INTS4 plays a key role in stabilizing nuclease domains and other components [22,32].

This study indicated that 14 INT subunits were all significantly increased in GC tissues compared with those normal gastric tissues based on TCGA databases. We further verified the 14 INT subunits mRNA expressions in single-cell sequencing dataset. The results showed that except for INTS1, INTS3, and INTS6, other 11 INT subunits in GC tissue cells were upregulated compared with normal tissue cells. To explore the cause of the abnormal expression of INT subunits in GC, we further assessed the genetic variations, constructed co-expression network, and applied functional enrichment analysis in GC. These results confirm that INT subunits were altered. Amplification and mutation of these subunits may be the main cause of their abnormal expression. Previous studies had demonstrated that INT subunits play important roles in RNA production and processing. In this study, GO analysis also revealed that INT subunits and those related genes were mainly involved in snRNA processing, INT, and DNA-directed 5′–3′ RNA polymerase activity. KEGG analysis of these subunits revealed RNA polymerase, nucleotide excision repair, and Huntington disease. Moreover, according to the results of TISIDB website analysis, there were close correlations between INT subunits and immune cell infiltration in GC. Our study also found that most of INT subunit expressions were positively related to the MSI, which indicated that INT subunits may play important roles in immune regulation in GC. Subsequently, MCPcounter algorithm showed that most INT subunit expressions were strongly positively related to neutrophil and endothelial cells. Neutrophils, as the most abundant leukocytes in peripheral blood, may influence tumor progression through the paracrine release of cytokines and chemokines with pro-tumor functions [33]. Also, it can form neutrophil extracellular traps to promote metastasis in GC patients and tumor-associated neutrophils were considered as a strong predictor for poor prognosis across human cancers [34,35]. The endothelial cells can line blood vessels and are especially prevalent in tumors [36]. The crosstalk between endothelial cells and tumor cells has been characterized as one of the key cell–cell interactions within the tumor microenvironment [37]. Endothelial cell-secreted epidermal growth factor induces epithelial-to-mesenchymal transition in head and neck cancer cells [37]. Tumor endothelial cells also can perform angiogenesis to support the growth, establishment, and dissemination of tumors to distant organs [38]. Our findings suggested that INT subunits involved in development of GC maybe be by influencing the proportion of neutrophil and endothelial cells.

## 5 Conclusion

In the present study, we systematically and comprehensively analyzed differential expression and mutation patterns, biological functions, protein–protein interactions, different prognostic values, and immune cell infiltration of INT subunits in GC patients based on public databases. Our analysis showed that most INT subunits were expressed at significantly elevated levels in GC tissues compared with normal tissues, suggesting that INT subunits may be potential predictors of prognosis for GC patients. Moreover, functional enrichment analysis indicated that differentially expressed INT subunits were mainly involved in snRNA processing, INT, and DNA-directed 5′–3′ RNA polymerase activity, and most INT subunit expressions were strongly positively related to MSI, neutrophil, and endothelial cells. Considering that INTS11 is the catalytic core subunit of the INT complex, knockdown or overexpression of the INTS11 subunit would influence the function of the INT complex. Therefore, we performed various experiments to assess the effect of INTS11 in GC cells. In conclusion, INT subunits could be effective markers with prognostic and expression significance for GC. Our results can help to better understand the pathogenesis of GC and develop more effective clinical treatments in the future.

## Appendix

**Table A1:** The sequence of qRT-PCR primers and shRNAs used in this study

Name			Sequence(5′–3′)	
**qRT-PCR primers**				
INTS11	Forward			GGAGCGCATGAACCTGAAG
	Reverse			GTCCAGGGGATGAACAGCTT
GAPDH	Forward			GTCGCCAGCCGAGCCACATC
	Reverse			CCAGGCGCCCAA TACGACCA
**shRNAs targeting INTS11**				
shRNA #1		sense		CCGGTCCTGGACTGTGTGATCATTA CTCGAGTAATGATCACACAGTC CAGGATTTTTG
		antisense		AATTCAAAAATCCTGGACTGTGTGATCATT ACTCGAGTAATGATCACACAGTCCAGGA
shRNA #2		sense		CCGGCCAGAAGAT CCGCAAGACTTTCTCGAGAAA GTCTTGCGGATCTTCTGGTTTTTG
		antisense		AATTCAAAAACCAGAAGA TCCGCAAGACTTTCTCGAGAAA GTCTTGCGGATCTTCTGG
